# Comparative clinical performance of robotic-assisted systems in spinal deformity surgery: focus on perioperative outcomes and pedicle screw accuracy

**DOI:** 10.1007/s11701-026-03410-9

**Published:** 2026-04-13

**Authors:** Yu Liu, Ruoyan Wang, Guohang Shen, Junlei Liao, Bo Mu, Yupei Dai, Nurul Azira Ismail

**Affiliations:** 1 Department of Pediatric Surgery, Affiliated Hospital of North Sichuan Medical College (Wenhua Road Campus), No. 63, Wenhua Road, Shunqing District, Nanchong City, 637000 Sichuan Province P.R. China; 2 School of Basic Medicine and Forensic Medicine, North Sichuan Medical College, No.234 Fujiang Road, Shunqing District, Nanchong, 637000 Sichuan China; 3 School of Graduate Studies Post Groduate Centre, Management and Science University (MSU), University Drive, Off Persiaran Olahraga, Section 13, Shah Alam, Selangor Malaysia; 4 Health Management Center of Affiliated Hospital of North Sichuan Medical College, Shunqing District, Nanchong City, 617000 Sichuan Province P.R. China; 5 Department of Clinical Medicine, Ningxia Medical University, Yinchuan, 750004 Ningxia Hui Autonomous Region PR China

**Keywords:** Spinal deformity, TiRobot, Mazor robotic system, Robot-assisted surgery, Systematic review, Meta-analysis

## Abstract

To compare the clinical effectiveness and safety of the TiRobot and Mazor robotic systems in spinal deformity surgery, with additional stratified analyses based on deformity type, including adolescent idiopathic scoliosis (AIS) and adult degenerative scoliosis (ADS). PubMed, Embase, Web of Science, and the Cochrane Library were systematically searched for comparative studies published up to February 1, 2026. Subgroup analyses were prespecified according to robotic platform (TiRobot vs. Mazor) and deformity type (AIS vs. ADS). Perioperative outcomes, radiographic parameters, pedicle screw accuracy, and safety endpoints were quantitatively synthesized. Compared with conventional techniques, robotic-assisted surgery was associated with prolonged operative time (MD = 29.64 min, P < 0.01), significantly improved pedicle screw accuracy (OR = 6.51, P < 0.01), and reduced radiation exposure (MD = − 5.27, P < 0.01), while no significant differences were observed in blood loss, hospital stay, or Cobb angle correction. Platform-based subgroup analyses indicated that TiRobot was associated with reduced intraoperative blood loss and shorter hospitalization, whereas Mazor demonstrated greater improvements in thoracic kyphosis and MT values. Disease-based subgroup analyses showed that operative time remained significantly longer in the AIS subgroup, whereas a shorter length of hospital stay was observed in ADS patients. No significant differences were detected in other outcomes across disease subtypes. Robotic assistance enhances pedicle screw accuracy and reduces radiation exposure. Both platform-specific and disease-specific differences exist in perioperative and radiographic outcomes, highlighting the importance of individualized surgical strategy based on both robotic system and deformity type.

**Supplementary Information:**

The online version contains supplementary material available at 10.1007/s11701-026-03410-9.

## Introduction

Spinal deformity represents a heterogeneous group of disorders characterized by three-dimensional spinal imbalance, encompassing adolescent idiopathic scoliosis, adult degenerative scoliosis, and congenital deformities. The defining feature of these conditions is abnormal spatial alignment of the spine across the coronal, sagittal, and axial planes, frequently accompanied by vertebral rotation, wedging, and pedicle dysplasia [1–3]. The etiology is multifactorial and may involve genetic predisposition, vertebral developmental anomalies, degenerative processes, and neuromuscular dysfunction. Progressive deformity not only results in visible trunk imbalance and functional limitation but may also lead to chronic pain, neurological compromise, and in severe cases, cardiopulmonary impairment, thereby substantially reducing quality of life [4, 5]. When conservative management fails or deformity progresses, surgical intervention becomes necessary to restore three-dimensional alignment, re-establish sagittal balance, and relieve neural compression. Contemporary corrective surgery relies fundamentally on accurate pedicle screw instrumentation to reconstruct spinal biomechanics and achieve stable fixation while minimizing perioperative morbidity [6, 7]. However, deformity-associated anatomical alterations—such as severe vertebral rotation, pedicle narrowing, and structural asymmetry—pose considerable technical challenges for safe and precise screw placement.

Conventional freehand pedicle screw insertion depends largely on the surgeon’s anatomical expertise and intraoperative fluoroscopic guidance. In cases with distorted or poorly defined anatomical landmarks, the risk of screw malposition increases substantially [8, 9]. Misplaced pedicle screws may result in neural or vascular injury, visceral damage, and occasionally necessitate revision surgery [10]. Moreover, repeated fluoroscopic confirmation contributes to cumulative radiation exposure for both patients and operating room staff. The combination of anatomical complexity and repeated imaging may also prolong operative time and increase intraoperative blood loss, thereby exacerbating physiological stress and potentially delaying postoperative recovery [11, 12]. These limitations underscore the need for more precise, reproducible, and radiation-sparing technologies to optimize surgical outcomes in spinal deformity correction.

With the rapid evolution of precision medicine and digital surgical technologies, robot-assisted systems have increasingly been incorporated into spinal deformity surgery. These platforms integrate three-dimensional imaging navigation, spatial registration algorithms, robotic arm stabilization, and real-time tracking technologies to facilitate preoperative trajectory planning and intraoperative execution. By translating preoperative imaging reconstruction into guided mechanical action, robotic systems are designed to enhance the spatial accuracy and reproducibility of pedicle screw placement [13, 14]. Compared with conventional freehand techniques, which rely heavily on surgeon experience and repeated fluoroscopic confirmation, robotic assistance theoretically mitigates tremor-related variability and subjective anatomical estimation, particularly in anatomically distorted deformities. Among currently available systems, the TiRobot platform, developed in China, and the internationally established Mazor robotic system are the most widely adopted in clinical practice. Although both aim to improve instrumentation precision, their technological architectures and workflow designs differ substantially. Variations exist in image registration strategies, degrees of freedom and stabilization of the robotic arm, trajectory planning algorithms, intraoperative adjustment mechanisms, and compatibility with imaging modalities such as O-arm or 3D C-arm systems. These structural and procedural differences may translate into heterogeneous clinical performance across key endpoints, including operative efficiency, perioperative tissue preservation, sagittal and coronal plane correction, and complication profiles.

Although existing studies consistently demonstrate the advantages of robotic assistance in enhancing pedicle screw accuracy and reducing radiation exposure, most investigations have evaluated each platform in isolation, limiting cross-system interpretation. Given the technological distinctions in image registration, trajectory planning algorithms, robotic arm stabilization, and intraoperative workflow, it is plausible that different platforms yield heterogeneous clinical performance in operative efficiency, perioperative tissue preservation, and radiographic correction outcomes. To date, however, no meta-analytic synthesis has systematically stratified results by robotic system to enable direct platform-level comparison. Therefore, in addition to comparing robotic-assisted and conventional techniques, the present study introduces a prespecified subgroup analysis based on deformity type (AIS vs. ADS), alongside platform-based stratification (TiRobot vs. Mazor). By integrating both technological and disease-level dimensions within a unified analytical framework, this study aims to provide a more comprehensive and clinically relevant evaluation of robotic-assisted spinal deformity surgery.

## Materials and methods

### Study design and registration

The study adhered to the PRISMA reporting framework, and the protocol was prospectively registered in PROSPERO (CRD420261321357) to enhance transparency and minimize selective reporting [15].

### Literature search strategy

A comprehensive search of PubMed, Embase, Web of Science, and the Cochrane Library was performed from database inception to February 1, 2026. Only English-language publications were considered. Detailed search syntax is provided in the Supplementary Material.

### Eligibility criteria

Studies were eligible if they:

1. compared robot-assisted techniques with conventional non-robotic methods in spinal deformity surgery, including randomized controlled trials and prospective or retrospective cohort studies;
2. enrolled patients diagnosed with spinal deformity (e.g., AIS, ADS, congenital deformities);
3. utilized either the TiRobot platform or the Mazor robotic system in the intervention arm, with freehand or fluoroscopy-guided techniques in the control arm;
4. reported at least one predefined endpoint, including perioperative outcomes (operative time, blood loss, length of stay), radiographic measures (Cobb angle change, LL, TK, MT value), pedicle screw accuracy (proportion of Gertzbein–Robbins Grade A + B screws), or safety outcomes (overall complications or radiation exposure).

Studies were excluded if they were non-comparative case series, reviews, prior meta-analyses, experimental studies, conference abstracts, dissertations, grey literature, lacked extractable data, or involved overlapping patient cohorts (in which case the most comprehensive dataset was retained).

### Quality assessment of included studies

Randomized trials were assessed using the Cochrane Risk of Bias tool [16]. Cohort studies were appraised using the Newcastle–Ottawa Scale (NOS).

### Statistical analysis

Analyses were performed using RevMan 5.4. Continuous variables were summarized as mean differences (MDs) with 95% confidence intervals (CIs), while dichotomous outcomes were expressed as odds ratios (ORs) with 95% CIs. Statistical heterogeneity was assessed using Cochran’s Q test and the I² statistic. A fixed-effects model was applied when I² < 50%; otherwise, a random-effects model was adopted. To explore potential sources of heterogeneity and improve the interpretability of pooled estimates, prespecified subgroup analyses were conducted according to two dimensions: robotic platform (TiRobot vs. Mazor), and deformity type (AIS vs. ADS). These subgroup analyses were applied across all major outcomes, including perioperative parameters, radiographic measurements, pedicle screw accuracy, and safety endpoints. Sensitivity analyses were performed by sequentially excluding individual studies to evaluate the robustness of the pooled results.

## Results

### Study selection and characteristics

**Fig. 1:**
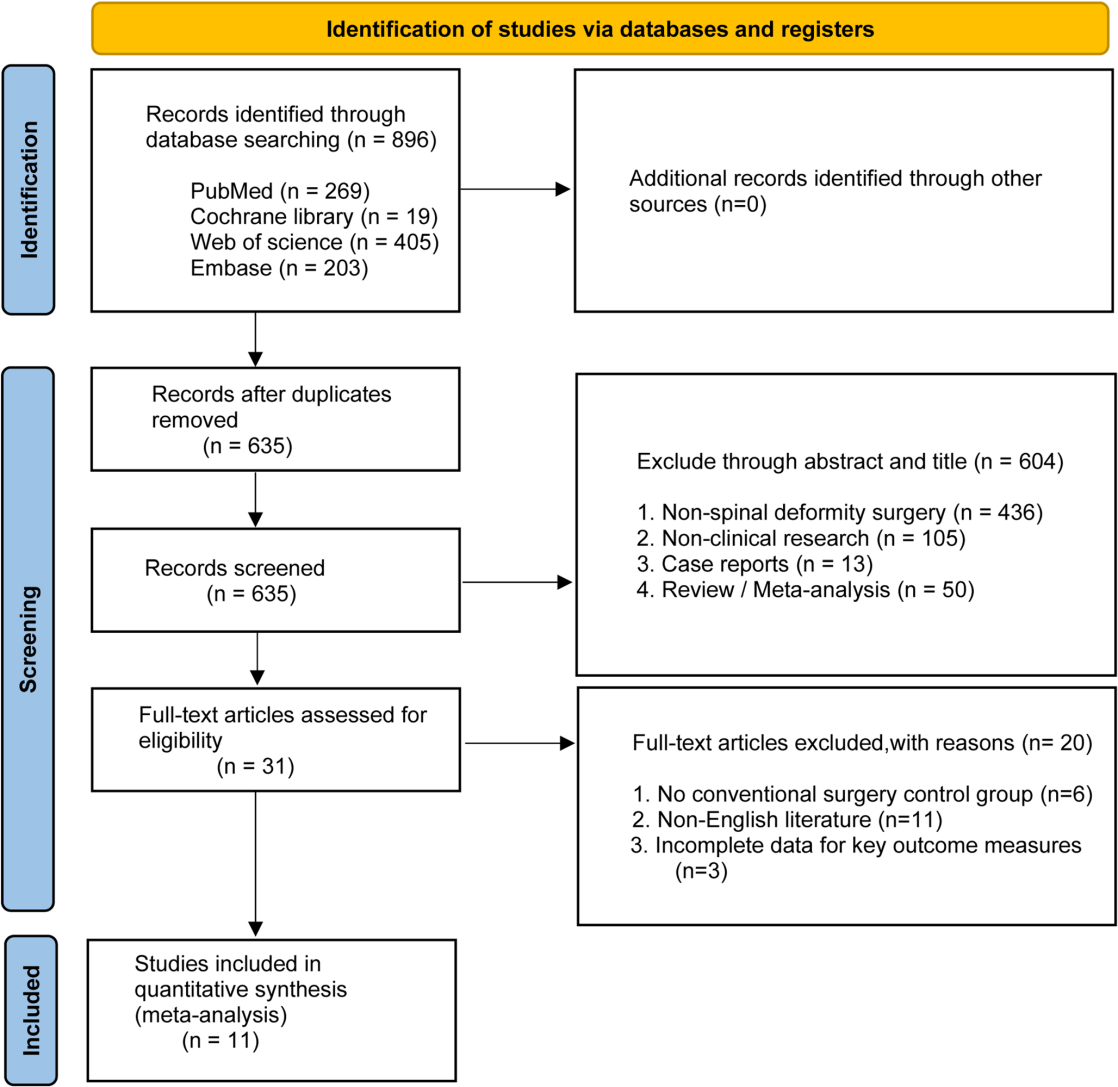
PRISMA flow diagram illustrating the study identification, screening, eligibility assessment, and final inclusion process

**Fig. 2:**
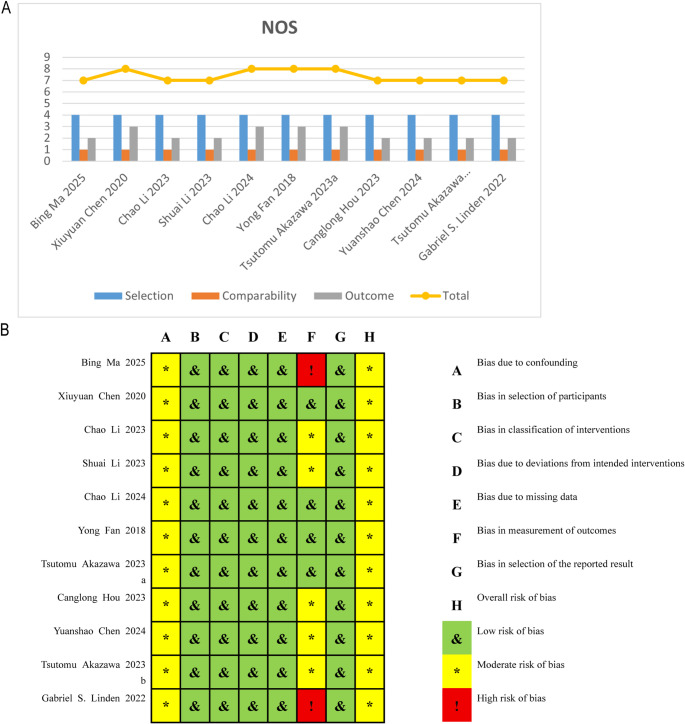
Methodological quality assessment of included studies. Non-randomized comparative studies were evaluated using the NOS, and risk of bias was assessed using the ROBINS-I tool

The initial database search retrieved 986 records. After removing 261 duplicates, 725 articles underwent title and abstract screening, resulting in exclusion of 604 studies. Full-text assessment led to the exclusion of an additional 20 articles. Ultimately, 11 comparative studies met inclusion criteria (Fig. 1) [17–27]. Five studies evaluated TiRobot and six assessed the Mazor system. All were non-randomized comparative designs. A total of eight studies focused on adolescent idiopathic scoliosis, whereas three studies investigated adult degenerative scoliosis. Study characteristics and baseline demographics are presented in Table 1, with outcome reporting summarized in Table 2. Methodological appraisal indicated overall moderate-to-high quality without critical bias (Fig. 2).

**Table 1:** Summary of patient demographics and methodological information in eligible comparative studies (RA: Robot-Assisted; CF: Conventional Freehand; N: Number of cases)

		Bing Ma	Xiuyuan Chen	Chao Li	Shuai Li	Chao Li	Yong Fan	Tsutomu Akazawa (a)	Canglong Hou	Yuanshao Chen	Tsutomu Akazawa (b)	Gabriel S. Linden
Publication year		2025	2020	2023	2023	2024	2018	2023	2023	2024	2023	2022
No. of patients	RA	22	31	32	40	92	83	13	45	31	11	30
(n)	CF	25	66	40	20	52	109	13	56	19	11	30
Gender (n) (female/male)												
	RA	12/10	12/19	6/26	25/15	24/68	48/35	11/2	32/13	26/5	10/1	23/7
	CF	8/17	25/41	10/30	8/12	17/35	65/44	11/2	38/18	14/5	9/2	23/7
Age (years) (mean ± SD)												
	RA	7.14 ± 2.10	69.8 ± 3.8	14.9 ± 3.1	13.20 ± 3.92	32.2 ± 22.8	61.6 ± 9.1	16.2 ± 2.5	14.69 ± 1.93	17.07 ± 5.44	16.4 ± 2.7	15 ± 2.01
	CF	7.28 ± 1.93	69.3 ± 5.1	15.4 ± 2.9	14.60 ± 2.97	29.1 ± 22.1	63.9 ± 8.4	15.8 ± 1.8	14.49 ± 2.01	17 ± 3.2	17.3 ± 2.6	15.3 ± 1.9
BMI, kg/m^2^ (mean ± SD)												
	RA	18.15 ± 2.79	24.5 ± 1.9	19.4 ± 2.8	22.43 ± 1.89	21.9 ± 2.8	25.8 ± 3.6	18.5 ± 2.6	NR	18.56 ± 3.34	18.9 ± 2.6	47.93 ± 35.03
	CF	18.81 ± 4.04	24.5 ± 2.1	20.4 ± 3.8	23.21 ± 1.62	22.4 ± 3.1	27.3 ± 3.9	18.6 ± 1.5	NR	17.72 ± 2.56	18.3 ± 1.4	72.16 ± 31.14
Lenke types (1/2/3/4/5/6) (n)												
	RA							7/3/0/0/3/0	22/8/4/1/7/3	8/10/3/1/9/0	5/3/0/0/3/0	NR
	CF							8/3/0/0/2/0	25/10/4/0/13/4	4/1/4/3/6/1	7/2/0/0/2/0	NR
Preoperative Cobb’s angle (°) (mean ± SD)												
	RA	37.05 ± 8.79	48.7 ± 6.4	48.1 ± 14.8	70.50 ± 12.89	48.7 ± 15.3	46 ± 8	18.4 ± 7.7	48.79 ± 7.03	61.62 ± 21.37	51.8 ± 7.2	NR
	CF	43.68 ± 7.56	47.4 ± 5.8	50.2 ± 20.7	65.20 ± 8.60	51.4 ± 14.4	49 ± 9	19.2 ± 4.1	47.14 ± 6.27	59.93 ± 25.63	50.7 ± 12.9	NR
Follow-up time (months) (mean ± SD)												
	RA	17.9 ± 3.8	11.1 ± 4.1	5.4 (mean)	14.8 ± 2.9		NR	1	2	35.22 ± 16.32	1	NR
	CF	18.4 ± 4.2	11.1 ± 3.4	5.4 (mean)	13.6 ± 1.2		NR	1	2	89.71 ± 37.65	1	NR
Study design		RCS	RCS	RCS	RCS	RCS	RCS	RCS	RCS	RCS	RCS	RCS
Type of robotic system		TiRobot	TiRobot	TiRobot	TiRobot	TiRobot	Mazor	Mazor	Mazor	Mazor	Mazor	Mazor

**Table 2:** Integrated analysis of operative variables and postoperative effectiveness outcomes reported in eleven studies

		Bing Ma	Xiuyuan Chen	Chao Li	Shuai Li	Chao Li	Yong Fan	Tsutomu Akazawa (a)	Canglong Hou	Yuanshao Chen	Tsutomu Akazawa (b)	Gabriel S. Linden
Operative time (min) (mean ± SD)												
	RA	166.32 ± 24.87	283.1 ± 30.8	393 ± 59.4	223.87 ± 19.82	408 ± 132	239 ± 52	295.8 ± 58.1	210.12 ± 11.78	506.44 ± 158.55	285.8 ± 60.2	235.57 ± 49.82
	CF	149.5 ± 13.72	291.9 ± 40	333.6 ± 59.4	215.73 ± 15.62	330 ± 96	228 ± 43	262.2 ± 44.1	179.07 ± 16.6	374.64 ± 120.95	281.7 ± 48.8	165.43 ± 42.03
Blood loss (ml) (mean ± SD)												
	RA		498.7 ± 96.3	515.4 ± 153.9	953.4 ± 120.4	627 ± 64	681 ± 277	293.1 ± 167	1063.07 ± 200.04	1591.92 ± 1476.66		228.88 ± 209.4
	CF		573 ± 78.1	540.8 ± 120.8	984.1 ± 176.9	643 ± 85	669 ± 250	335.2 ± 367.4	804.56 ± 137.17	1088.21 ± 800.97		163.55 ± 107.42
Length of hospital stay (days) (mean ± SD)												
	RA		12.8 ± 4.5	9.13 ± 2.61	10.12 ± 2.43	14.5 ± 3.2	9.3 ± 2.2			7 ± 1.6		3.36 ± 0.78
	CF		13.7 ± 4.6	9.52 ± 3.49	9.86 ± 2.67	16.1 ± 3.4	9.5 ± 2			8 ± 1.6		3.36 ± 0.78
Change in Cobb angle (mean ± SD)												
	RA	8.67 ± 7.27	10.6 ± 1.9	16.6 ± 8.5		25.9 ± 20.1	11 ± 7	18.4 ± 7.7	13.74 ± 5.11	22.37 ± 8.86	49.7 ± 6.7	
	CF	8.84 ± 6.65	10.9 ± 1.9	21.8 ± 17.6		20.6 ± 19.5	12 ± 9	19.2 ± 4.1	12.97 ± 4.56	26.87 ± 23.79	52.4 ± 9.6	
LL	RA		51.8 ± 4.4		43.62 ± 6.33		23 ± 11		38.74 ± 7.05			
(mean ± SD)	CF		50.3 ± 5		43.72 ± 5.6		22 ± 13		35.95 ± 8.52			
TK	RA					23.4 ± 13.1		21.4 ± 6.1		19.04 ± 8.01		
(mean ± SD)	CF					24.1 ± 13.1		25.1 ± 4.6		28.67 ± 21.71		
MT	RA							17.2 ± 5			42.3 ± 14.2	
(mean ± SD)	CF							19.5 ± 3.4			49.6 ± 12.6	
Proportion of Gertzbein–Robbins Grade A + B screws (n)												
	RA	145/146	373/378	606/627				180/183			246/250	
	CF	134/151	725/786	624/776				188/196			454/491	
Complications (n)	RA		1	0		2	6		6			
	CF		2	2		3	9		11			
Radiation exposure dose (mean ± SD)												
	RA				412 ± 147		0.41 ± 0.39					
	CF				385 ± 150		5.68 ± 2.66					

### Perioperative outcomes

1. **Operative Time.**

**Fig. 3:**
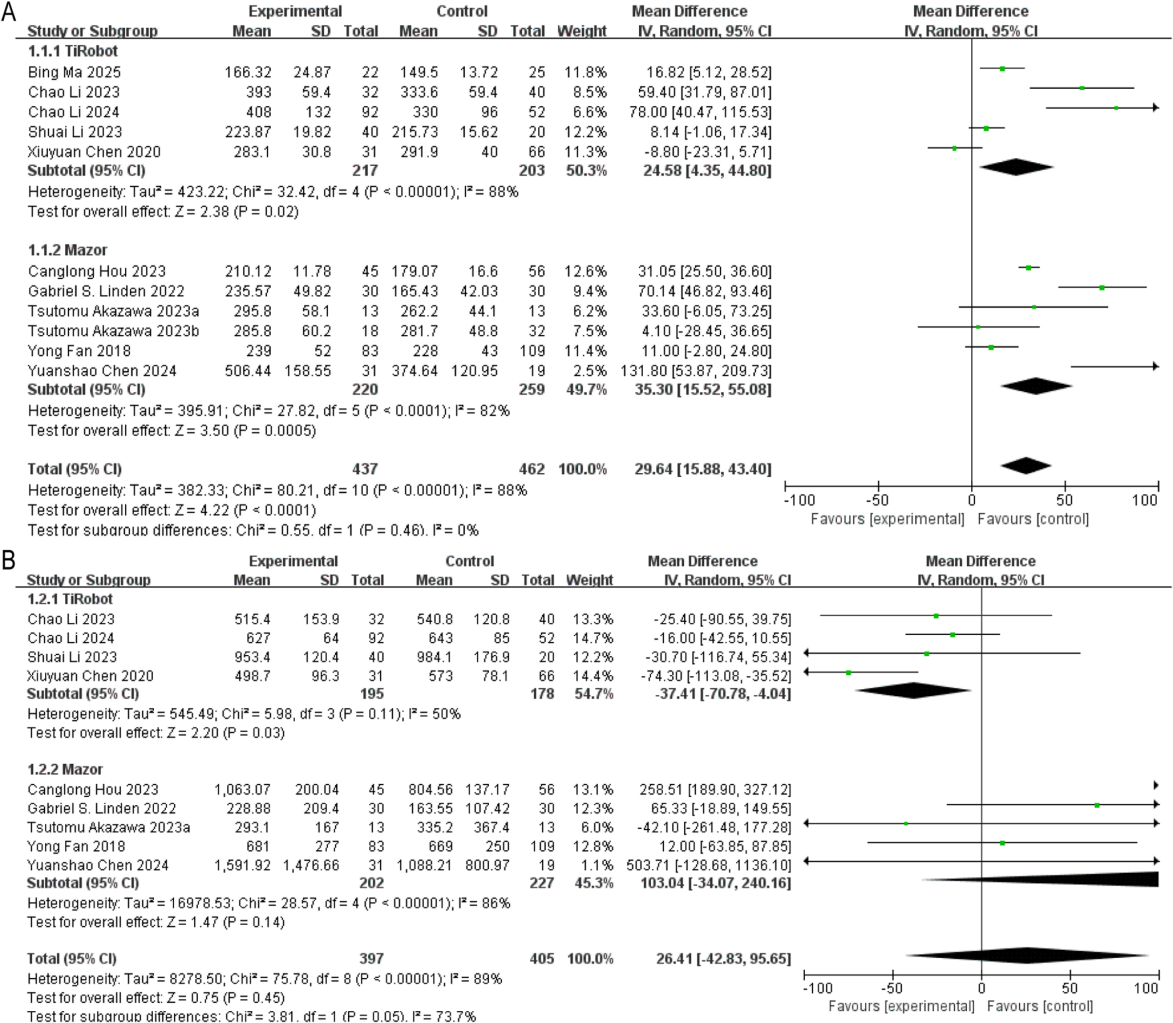
Forest plots of perioperative outcomes comparing robot-assisted and conventional surgery. (**A**) Operative time; (**B**) Intraoperative blood loss

Across all 11 studies, robot-assisted procedures required additional operative time compared with conventional techniques (MD = 29.64 min, 95% CI: 15.88–43.40, *P* < 0.01) (Fig. 3A).

This pattern was observed in both platforms: TiRobot (*n* = 5) demonstrated an increase of 24.58 min (95% CI: 4.35–44.80, *P* < 0.05), while Mazor (*n* = 6) showed a prolongation of 35.30 min (95% CI: 15.52–55.08, *P* < 0.01). In the AIS subgroup, operative time was also significantly longer in the robotic group (MD = 33.64 min, 95% CI: 18.47–48.81, *P* < 0.01) (Supplementary Fig. 1A).

(2) **Intraoperative Blood Loss**

Nine studies contributed data. When aggregated, no significant overall difference emerged (MD = 26.41, 95% CI: −42.83 to 95.65, *P* > 0.05) (Fig. 3B).

However, stratification revealed reduced blood loss with TiRobot (MD = − 37.41, 95% CI: −70.78 to − 4.04, *P* < 0.05), whereas the Mazor subgroup showed no significant change (MD = 103.04, 95% CI: −34.07 to 240.16, *P* > 0.05).

No statistically significant differences were observed in either the AIS or ADS subgroups (Supplementary Fig. 1B).

(3) **Length of Hospital Stay**

**Fig. 4:**
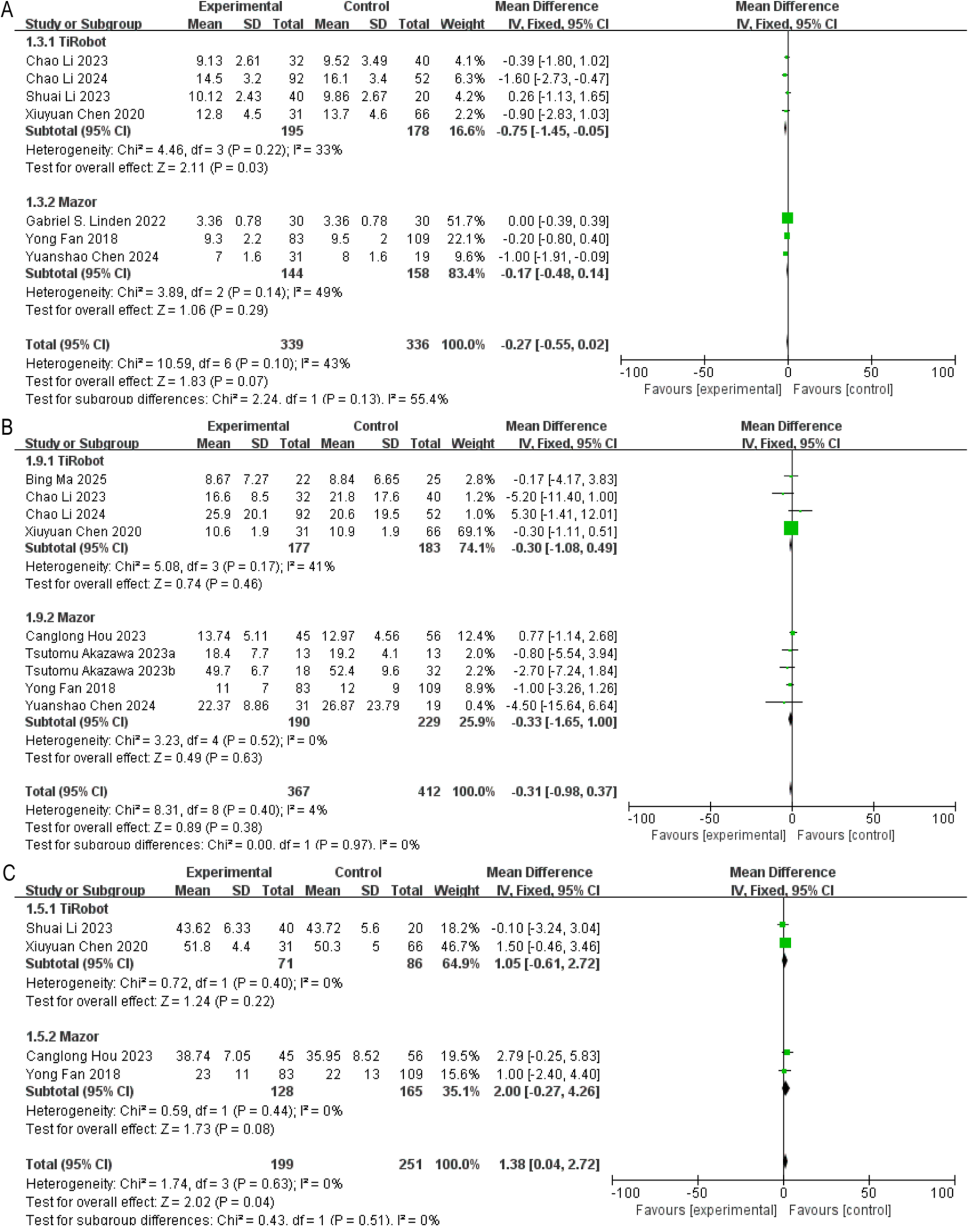
Forest plots of hospitalization and selected radiographic outcomes. (**A**) Length of hospital stay; (**B**) Change in Cobb angle; (**C**) Improvement in lumbar lordosis

Seven studies examined hospital stay. The pooled estimate indicated no statistically significant overall difference (MD = − 0.27 days, 95% CI: −0.55 to 0.02, *P* > 0.05) (Fig. 4A).

Platform-specific analysis showed shorter hospitalization in the TiRobot subgroup (MD = − 0.75 days, 95% CI: −1.14 to − 0.05, *P* < 0.05), whereas no significant reduction was observed with Mazor (MD = − 0.17 days, 95% CI: −0.48 to 0.14, *P* > 0.05).

In the ADS subgroup, hospitalization duration was significantly shorter in the robotic group (MD = − 0.54 days, 95% CI: −1.05 to − 0.02, *P* < 0.05) (Supplementary Fig. 1C).

### Radiographic outcomes

1. **Change in Cobb Angle**

Nine studies assessed Cobb angle change. The pooled comparison showed no significant difference (MD = − 0.31, 95% CI: −0.98 to 0.37, *P* > 0.05). Neither robotic platform demonstrated superiority in subgroup analysis (Fig. 4B).

Subgroup analyses based on AIS and ADS also showed no significant differences (Supplementary Fig. 2A).

(2) **Lumbar Lordosis (LL)**

Four studies reported LL improvement. The combined analysis indicated a modest but statistically significant greater increase in LL in the robotic cohort (MD = 1.38, 95% CI: 0.04 to 2.72, *P* < 0.05) (Fig. 4C). Platform-level stratification did not yield statistically significant differences individually.

However, subgroup analyses did not show significant differences when stratified by platform or disease type (Supplementary Fig. 2B).

(3) **Thoracic Kyphosis (TK)**

**Fig. 5:**
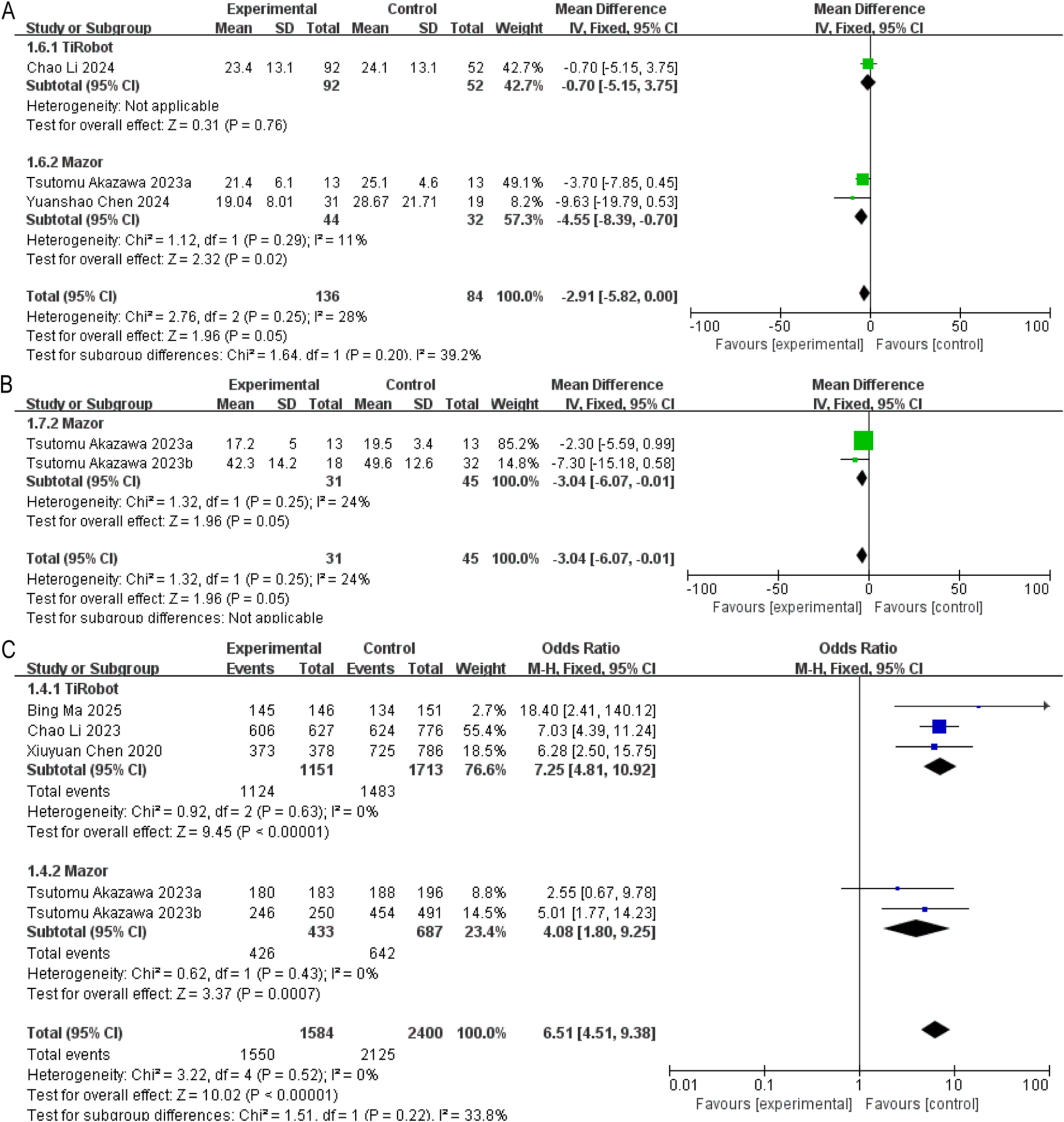
Forest plots of radiographic parameters and pedicle screw accuracy. (**A**) Thoracic kyphosis; (**B**) MT value; (**C**) Proportion of Gertzbein–Robbins Grade A + B screws

Three studies examined TK. The overall estimate approached statistical significance (MD = − 2.91, 95% CI: −5.82 to 0.00, *P* = 0.05). Only the Mazor subgroup permitted pooling (*n* = 2), demonstrating significantly lower TK values (MD = − 4.55, 95% CI: −8.39 to − 0.70, *P* < 0.05) (Fig. 5A).

This finding was also observed in the AIS subgroup (Supplementary Fig. 2C).

(4) **MT Value**

Two Mazor-based studies evaluated MT. The pooled estimate indicated lower MT values with robotic assistance (MD = − 3.04, 95% CI: −6.07 to − 0.01, *P* = 0.05) (Fig. 5B). No TiRobot studies reported this parameter.

### Pedicle screw placement accuracy

Five studies reported the proportion of Gertzbein–Robbins Grade A + B screws. The overall odds ratio strongly favored robotic assistance (OR = 6.51, 95% CI: 4.51–9.38, *P* < 0.01) (Fig. 5C). This advantage persisted in both TiRobot (OR = 7.25, 95% CI: 4.81–10.92, *P* < 0.01) and Mazor (OR = 4.08, 95% CI: 1.80–9.25, *P* < 0.01) subgroups.

In the AIS subgroup, the robotic group also showed higher accuracy (OR = 6.56, 95% CI: 4.41–9.76, *P* < 0.05) (Supplementary Fig. 3A).

### Safety outcomes

1. **Overall Complication Rate**

**Fig. 6:**
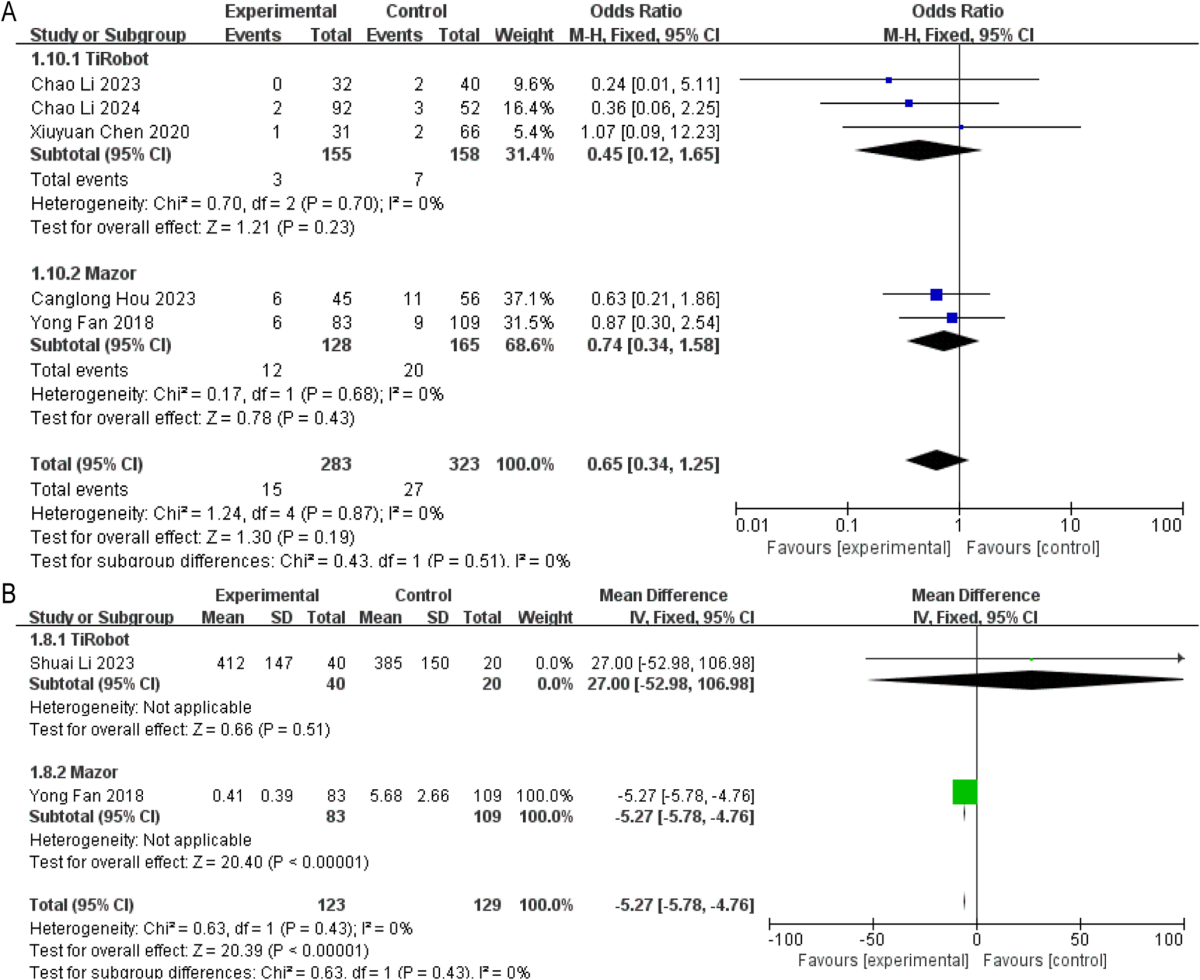
Forest plots of safety outcomes. (**A**) Overall complication rate; (**B**) Intraoperative radiation exposure

Across five studies, no statistically significant difference in complication rates was observed (OR = 0.65, 95% CI: 0.34–1.25, *P* > 0.05) (Fig. 6A).

No significant differences were found in AIS or ADS subgroups (Supplementary Fig. 3B).

(2) **Radiation Exposure**

Two studies reported radiation data. Robotic assistance significantly reduced radiation exposure (MD = − 5.27, 95% CI: −5.78 to − 4.76, *P* < 0.01) (Fig. 6B).

### Sensitivity analysis

Sequential exclusion of individual studies did not materially alter pooled estimates for operative time, blood loss, screw accuracy, or complications, supporting the stability of the findings.

## Discussion

This systematic review and meta-analysis synthesized current comparative evidence on the clinical performance of two major robotic platforms—TiRobot and the Mazor robotic system—in spinal deformity surgery. By implementing a prespecified platform-stratified analytical framework, this study extends beyond conventional pooled comparisons and provides a nuanced evaluation of platform-dependent performance characteristics. The results demonstrate that robotic assistance offers consistent improvements in pedicle screw placement accuracy and radiation reduction, whereas perioperative efficiency and radiographic correction profiles exhibit system-specific patterns. Notably, prolonged operative duration emerged as a reproducible finding across both platforms.

### Interpretation of perioperative findings

The observed increase in operative time across robotic cohorts likely reflects the procedural demands intrinsic to image-guided workflows rather than technological inefficiency. Robotic integration necessitates preoperative image acquisition, spatial registration, trajectory planning, and mechanical calibration, particularly critical in anatomically distorted deformities characterized by vertebral rotation and pedicle dysplasia. These preparatory steps introduce time overhead but enhance geometric reproducibility and reduce reliance on intraoperative anatomical estimation. Another important factor contributing to prolonged operative time is the learning curve associated with robotic-assisted surgery. During the early adoption phase, typically encompassing the initial 20–30 cases, surgeons and operating teams must adapt to system setup, image registration, trajectory planning, and workflow integration. These factors may substantially increase operative duration compared to both conventional techniques and later-stage robotic practice. Notably, most included studies did not stratify outcomes according to surgeon experience or learning curve phase, limiting the ability to distinguish between early-phase inefficiency and mature procedural performance. Therefore, the observed increase in operative time may partially reflect transitional learning effects rather than intrinsic limitations of robotic systems.

Subgroup analysis revealed that TiRobot-assisted procedures were associated with reduced intraoperative blood loss and shorter hospitalization, whereas comparable benefits were not statistically evident in Mazor cohorts. This discrepancy may be partially explained by workflow architecture and case complexity distribution. TiRobot integrates real-time optical tracking with minimally invasive trajectory execution, potentially limiting paraspinal soft-tissue dissection and cumulative manipulation. Conversely, Mazor-based studies may have included proportionally more complex or multi-segment deformities, thereby attenuating measurable perioperative gains. These findings underscore the importance of contextual interpretation when comparing heterogeneous robotic ecosystems [27, 28]. An important consideration in interpreting perioperative outcomes is the potential presence of directional selection bias inherent to non-randomized surgical studies. In routine clinical practice, robotic-assisted techniques are often preferentially applied to more complex deformities, including rigid curves, multi-level reconstructions, or revision cases, whereas conventional freehand approaches may be reserved for less complex and more flexible deformities. This imbalance in baseline case complexity may confound comparisons and partially explain the observed prolongation in operative time and the absence of significant reductions in intraoperative blood loss in the robotic cohorts. Therefore, these findings should not be interpreted solely as indicators of procedural inefficiency but rather as reflecting, at least in part, differences in surgical case mix and indication.

### Radiographic correction and sagittal alignment

Radiographic outcomes demonstrated modest yet clinically relevant signals favoring robotic assistance in sagittal-plane parameters. The pooled improvement in lumbar lordosis and the reduction in thoracic kyphosis suggest that robotic trajectory precision may contribute indirectly to sagittal balance restoration. Although a statistically significant improvement in lumbar lordosis was observed, the absolute magnitude of change was minimal and unlikely to be clinically meaningful in the context of global spinal deformity correction. Furthermore, the limited number of studies reporting sagittal parameters (LL, TK, MT) restricts the strength of evidence. Therefore, the role of robotic assistance in sagittal alignment reconstruction remains inconclusive and should be interpreted cautiously.

Importantly, no significant intergroup differences were detected in Cobb angle correction. Coronal alignment is multifactorial and influenced by osteotomy strategy, rod contouring, fusion length, and deformity rigidity, parameters not directly governed by screw trajectory accuracy alone. Thus, while robotics enhances instrumentation precision, its incremental impact on global coronal correction may be inherently constrained. Future research focusing on severe or rigid deformities may better delineate the boundaries of robotic contribution to three-dimensional structural restoration [29, 30].

### Instrumentation accuracy and safety implications

Pedicle screw accuracy represents the most robust and consistent advantage of robotic assistance. The substantial increase in Gertzbein–Robbins Grade A + B screw proportion across both platforms substantiates the mechanistic premise of robotic stabilization and spatial navigation. Although the TiRobot subgroup demonstrated numerically higher effect estimates, sample-size limitations preclude definitive comparative conclusions [31].

Radiation exposure was significantly reduced in robotic cohorts, consistent with workflow paradigms emphasizing pre-acquired imaging and trajectory planning over repeated fluoroscopic confirmation. This reduction carries implications not only for patient safety but also for occupational health protection of surgical teams.

In contrast, no statistically significant reduction in overall complication rates was identified. This finding may reflect limited statistical power, low baseline complication incidence, and heterogeneity in deformity severity. Consequently, while robotic systems enhance technical precision, translation into measurable clinical complication reduction remains to be conclusively established.

### Strengths and limitations

#### Strengths

Several methodological strengths enhance the credibility and clinical relevance of this study. First, this is the first meta-analysis to incorporate prespecified subgroup stratification by robotic platform, enabling platform-level performance differentiation rather than treating robotic assistance as a homogeneous intervention. Second, protocol registration in PROSPERO and adherence to PRISMA standards improve methodological transparency and reduce selective reporting bias. Third, comprehensive evaluation of perioperative, radiographic, technical accuracy, and safety endpoints allows multidimensional assessment of robotic performance within a unified analytical framework. Finally, sensitivity analyses demonstrated stability of pooled estimates, supporting robustness of the findings. Importantly, by incorporating both platform-specific and disease-specific subgroup analyses (AIS vs. ADS), this study attempts to address key sources of clinical heterogeneity and provide a more nuanced interpretation of robotic-assisted spinal deformity surgery.

#### Limitations

Nevertheless, several limitations warrant cautious interpretation. All included studies were non-randomized comparative cohorts, introducing inherent risks of selection bias, confounding, and unmeasured baseline heterogeneity. Differences in deformity severity, fusion levels, surgical expertise, and institutional protocols may have influenced outcomes. Second, certain endpoints—particularly MT value and radiation exposure—were reported in a limited number of studies, restricting statistical power and precision of effect estimation. Additionally, sagittal alignment parameters were reported in only a small number of studies, limiting statistical power and reducing confidence in conclusions related to alignment reconstruction. Third, follow-up duration was generally short, precluding evaluation of long-term functional outcomes, implant longevity, and adjacent segment pathology. Fourth, cost-effectiveness, learning curve dynamics, and training standardization were not systematically assessed, although these factors critically influence real-world implementation. Finally, conclusions are restricted to TiRobot and Mazor systems and may not be generalizable to other robotic platforms.

Future multicenter randomized trials with standardized deformity stratification, long-term follow-up, and health-economic modeling are required to more definitively characterize the value proposition of robotic spinal surgery.

## Conclusion

Current evidence indicates that robotic assistance in spinal deformity surgery primarily enhances pedicle screw accuracy and reduces intraoperative radiation exposure, with consistent effects observed for both TiRobot and Mazor systems. These benefits are accompanied by prolonged operative time. Platform-specific differences were noted in selected perioperative and sagittal parameters, yet no reduction in overall complication rates was demonstrated. Robotic systems should therefore be regarded as precision-enhancing technologies rather than universal substitutes for conventional techniques. Platform selection should be individualized according to deformity characteristics and surgical context.

## Supplementary Information

Below is the link to the electronic supplementary material.

Supplementary Material 1

Supplementary Material 2: Forest plots of perioperative outcomes stratified by deformity etiology (AIS vs. ADS) comparing robot-assisted and conventional surgery. (A) Operative time; (B) Intraoperative blood loss; (C) Length of hospital stay.

Supplementary Material 3: Forest plots of radiographic outcomes stratified by deformity etiology (AIS vs. ADS) comparing robot-assisted and conventional techniques. (A) Change in Cobb angle; (B) Lumbar lordosis; (C) Thoracic kyphosis.

Supplementary Material 4: Forest plots of technical and safety outcomesstratified by deformity etiology (AIS vs. ADS) comparing robot-assisted andconventional surgery. (A) Proportion of Gertzbein–Robbins Grade A+B screws;(B) Overall complication rate.

## Data Availability

No datasets were generated or analysed during the current study.
