# Supplementary material for: Comparative clinical performance of robotic-assisted systems in spinal deformity surgery: focus on perioperative outcomes and pedicle screw accuracy
Source: J Robot Surg. 2026 Apr 13;20(1):432. doi: 10.1007/s11701-026-03410-9 (PMC13070983; doi:10.1007/s11701-026-03410-9)
Supplement: Supplementary file 1 — Supplementary Material 1 [file 11701_2026_3410_MOESM1_ESM.docx]

**1. Pubmed**

(((Scoliosis[Title/Abstract]) OR (Spinal deformity[Title/Abstract])) OR (Spine deformity[Title/Abstract])) AND ((((((((((((((((((((((((((((((((((((((((Robot) OR (Robots)) OR (Robotic)) OR (Robotics)) OR (Remote Operation)) OR (Operations, Remote)) OR (Operation, Remote)) OR (Remote Operations)) OR (Robotic Surgical Procedures)) OR (Robot-Enhanced)) OR (Robot-Assisted)) OR (Procedures, Robotic Surgical)) OR (Robotic-Assisted)) OR (Procedure, Robotic Surgical)) OR (Remote Operations (Robotics))) OR (Operation, Remote (Robotics))) OR (Operations, Remote (Robotics))) OR (Remote Operation (Robotics))) OR (Telerobotics)) OR (Soft Robotics)) OR (Robotic, Soft)) OR (Soft Robotic)) OR (Assistive Robot)) OR (Socially Assistive Robots)) OR (Assistive Robot, Socially)) OR (Robot, Socially Assistive)) OR (Socially Assistive Robot)) OR (Social Robots)) OR (Robot, Social)) OR (Social Robot)) OR (Humanoid Robots)) OR (Humanoid Robot)) OR (Robot, Humanoid)) OR (Companion Robots)) OR (Companion Robot)) OR (Robot, Companion)) OR ("Robotics"[Mesh])))))

**2. Cochrane**

| #3 | #1 and #2 |
| --- | --- |
| #2 | (Scoliosis):ti,ab,kw OR (Spinal deformity):ti,ab,kw OR (Spine deformity):ti,ab,kw |
| #1 | (Robot):ti,ab,kw OR (Robots):ti,ab,kw OR (Robotic):ti,ab,kw OR (Robotics):ti,ab,kw OR (Remote Operation):ti,ab,kw OR (Operations, Remote):ti,ab,kw OR (Operation, Remote):ti,ab,kw OR (Remote Operations):ti,ab,kw OR (Robotic Surgical Procedures):ti,ab,kw OR (Robot-Enhanced):ti,ab,kw OR (Robot-Assisted):ti,ab,kw OR (Procedures, Robotic Surgical):ti,ab,kw OR (Robotic-Assisted):ti,ab,kw OR (Procedure, Robotic Surgical):ti,ab,kw OR (Remote Operations (Robotics)):ti,ab,kw OR (Operation, Remote (Robotics)):ti,ab,kw OR (Operations, Remote (Robotics)):ti,ab,kw OR (Remote Operation (Robotics)):ti,ab,kw OR (Telerobotics):ti,ab,kw OR (Soft Robotics):ti,ab,kw OR (Robotic, Soft):ti,ab,kw OR (Soft Robotic):ti,ab,kw OR (Assistive Robot):ti,ab,kw OR (Socially Assistive Robots):ti,ab,kw OR (Assistive Robot, Socially):ti,ab,kw OR (Robot, Socially Assistive):ti,ab,kw OR (Socially Assistive Robot):ti,ab,kw OR (Social Robots):ti,ab,kw OR (Robot, Social):ti,ab,kw OR (Social Robot):ti,ab,kw OR (Humanoid Robots):ti,ab,kw OR (Humanoid Robot):ti,ab,kw OR (Robot, Humanoid):ti,ab,kw OR (Companion Robots):ti,ab,kw OR (Companion Robot):ti,ab,kw OR (Robot, Companion):ti,ab,kw |

**3. Web of science**

| #3 | #1 and #2 |
| --- | --- |
| #2 | TS=(Scoliosis or Spinal deformity or Spine deformity) |
| #1 | TS=(Robot or Robots or Robotic or Robotics or Remote Operation or Operations, Remote or Operation, Remote or Remote Operations or Robotic Surgical Procedures or Robot-Enhanced or Robot-Assisted or Procedures, Robotic Surgical or Robotic-Assisted or Procedure, Robotic Surgical or Remote Operations (Robotics) or Operation, Remote (Robotics) or Operations, Remote (Robotics) or Remote Operation (Robotics) or Telerobotics or Soft Robotics or Robotic, Soft or Soft Robotic or Assistive Robot or Socially Assistive Robots or Assistive Robot, Socially or Robot, Socially Assistive or Socially Assistive Robot or Social Robots or Robot, Social or Social Robot or Humanoid Robots or Humanoid Robot or Robot, Humanoid or Companion Robots or Companion Robot or Robot, Companion) |

**4. Embase**

| #3 | #1 and #2 |
| --- | --- |
| #2 | 'Scoliosis':ab,ti OR 'Spinal deformity':ab,ti OR 'Spine deformity':ab,ti |
| #1 | 'robot':ab,ti OR 'robots':ab,ti OR 'robotic':ab,ti OR 'robotics':ab,ti OR 'remote operation':ab,ti OR 'operations, remote':ab,ti OR 'operation, remote':ab,ti OR 'remote operations':ab,ti OR 'robotic surgical procedures':ab,ti OR 'robot-enhanced':ab,ti OR 'robot-assisted':ab,ti OR 'procedures, robotic surgical':ab,ti OR 'robotic-assisted':ab,ti OR 'procedure, robotic surgical':ab,ti OR 'remote operations (robotics)':ab,ti OR 'operation, remote (robotics)':ab,ti OR 'operations, remote (robotics)':ab,ti OR 'remote operation (robotics)':ab,ti OR 'telerobotics':ab,ti OR 'soft robotics':ab,ti OR 'robotic, soft':ab,ti OR 'soft robotic':ab,ti OR 'assistive robot':ab,ti OR 'socially assistive robots':ab,ti OR 'assistive robot, socially':ab,ti OR 'robot, socially assistive':ab,ti OR 'socially assistive robot':ab,ti OR 'social robots':ab,ti OR 'robot, social':ab,ti OR 'social robot':ab,ti OR 'humanoid robots':ab,ti OR 'humanoid robot':ab,ti OR 'robot, humanoid':ab,ti OR 'companion robots':ab,ti OR 'companion robot':ab,ti OR 'robot, companion':ab,ti |
