# Supplementary figures and images for: Comparative clinical performance of robotic-assisted systems in spinal deformity surgery: focus on perioperative outcomes and pedicle screw accuracy
Source: J Robot Surg. 2026 Apr 13;20(1):432. doi: 10.1007/s11701-026-03410-9 (PMC13070983; doi:10.1007/s11701-026-03410-9)

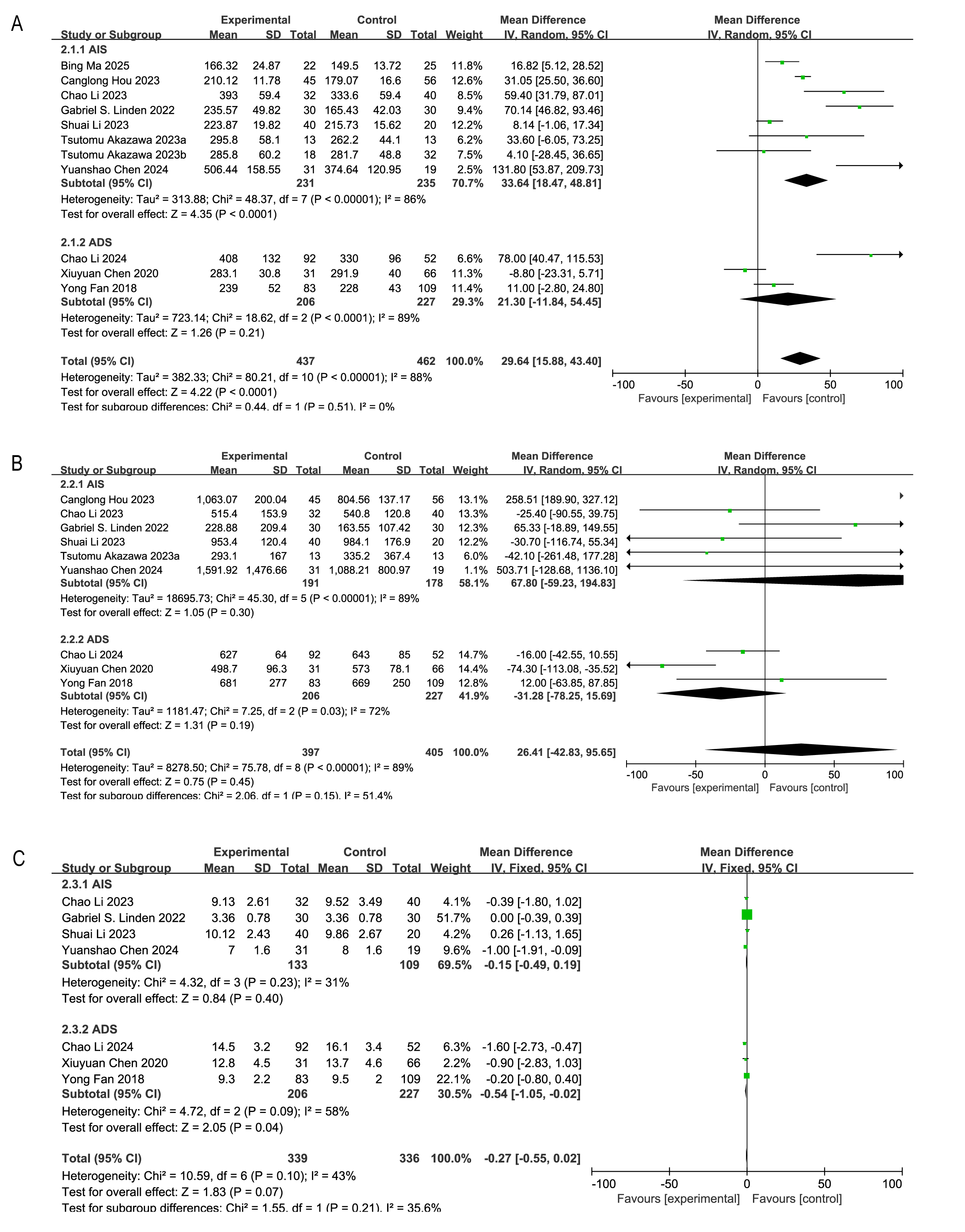

Supplement: Supplementary file 2 — Supplementary Material 2: Forest plots of perioperative outcomes stratified by deformity etiology (AIS vs. ADS) comparing robot-assisted and conventional surgery. (A) Operative time; (B) Intraoperative blood loss; (C) Length of hospital stay. [file 11701_2026_3410_MOESM2_ESM.tif]

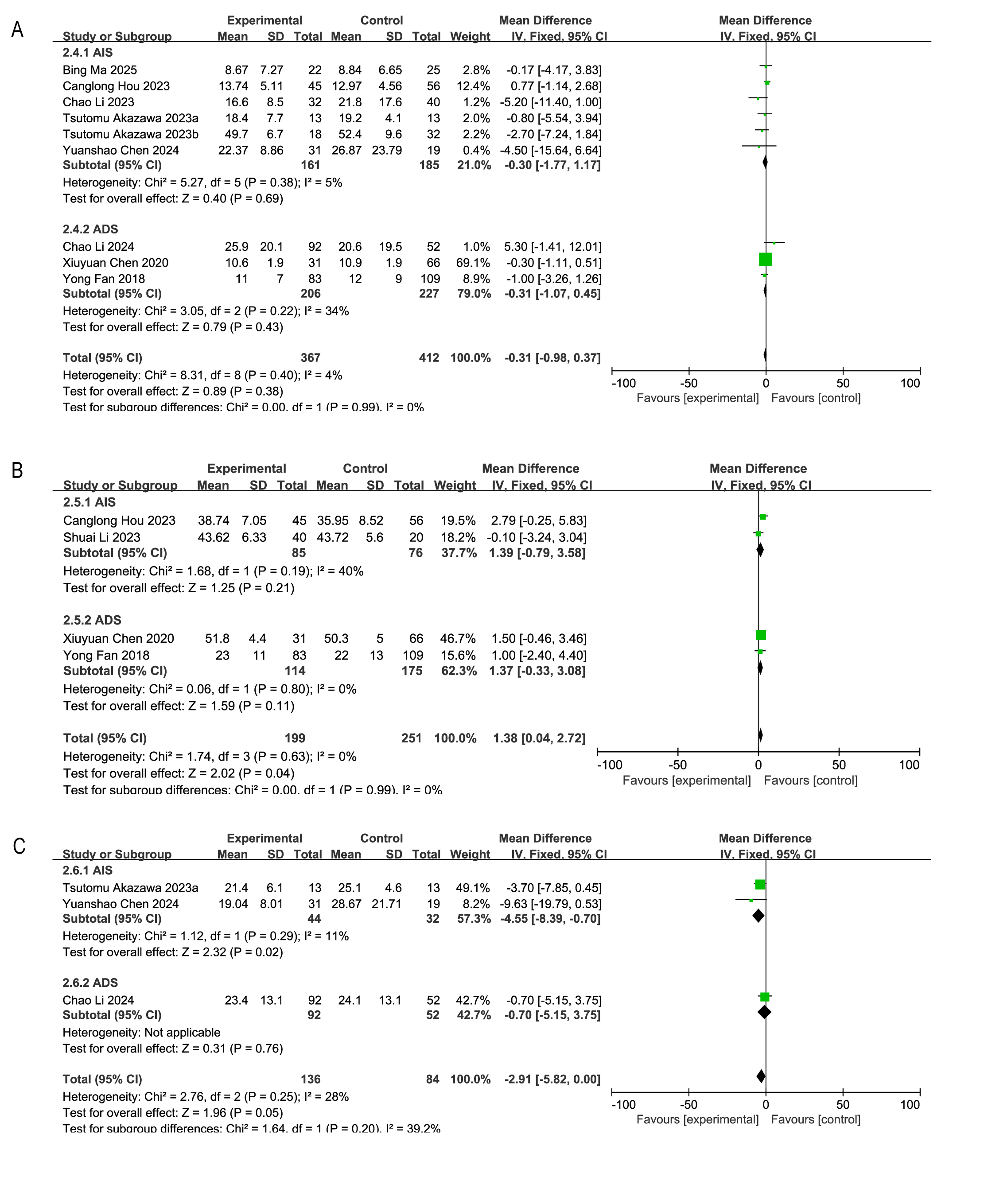

Supplement: Supplementary file 3 — Supplementary Material 3: Forest plots of radiographic outcomes stratified by deformity etiology (AIS vs. ADS) comparing robot-assisted and conventional techniques. (A) Change in Cobb angle; (B) Lumbar lordosis; (C) Thoracic kyphosis. [file 11701_2026_3410_MOESM3_ESM.tif]

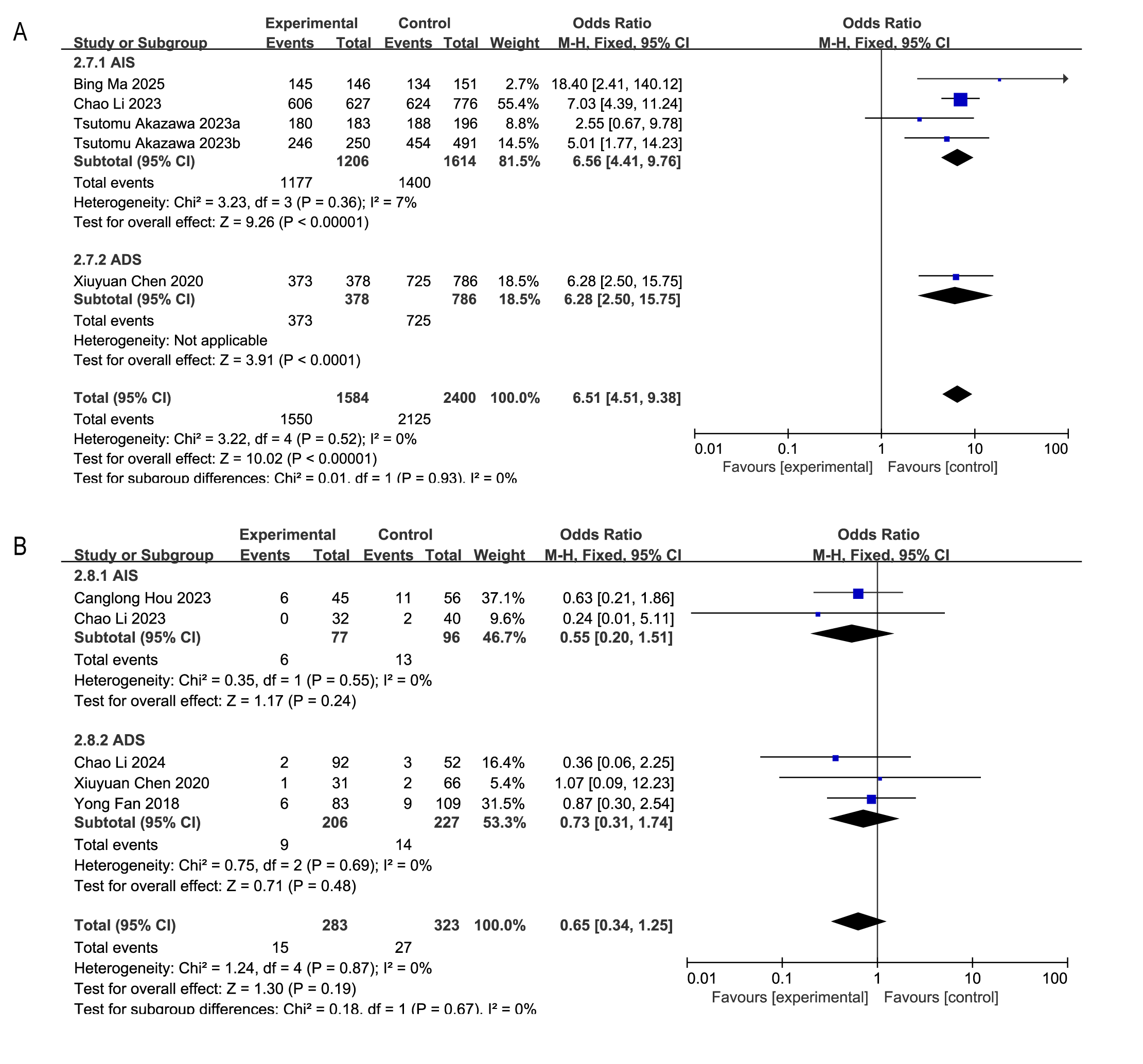

Supplement: Supplementary file 4 — Supplementary Material 4: Forest plots of technical and safety outcomesstratified by deformity etiology (AIS vs. ADS) comparing robot-assisted andconventional surgery. (A) Proportion of Gertzbein–Robbins Grade A+B screws;(B) Overall complication rate. [file 11701_2026_3410_MOESM4_ESM.tif]
